# Diagnostic Accuracy of the ADNEX Model for Ovarian Cancer at the 15% Cut-Off Value: A Systematic Review and Meta-Analysis

**DOI:** 10.3389/fonc.2021.684257

**Published:** 2021-06-17

**Authors:** Xiaotong Huang, Ziwei Wang, Meiqin Zhang, Hong Luo

**Affiliations:** ^1^ Department of Ultrasound, West China Second University Hospital, Sichuan University, Chengdu, China; ^2^ Key Laboratory of Birth Defects and Related Diseases of Women and Children, Sichuan University, Chengdu, China

**Keywords:** ovarian cancer, ADNEX model, ultrasonography, diagnostic accuracy, preoperative identification

## Abstract

**Objectives:**

To evaluate the diagnostic accuracy of the ADNEX model for ovarian cancer at the 15% cut-off value.

**Methods:**

Studies on the identified diagnosis of the ADNEX model for ovarian cancer published in PubMed, Embase, the Cochrane Library and Web of Science databases from January 1st, 2014 to February 20th, 2021 were searched. Two researchers independently screened the retrieved studies and extracted the basic features and parameter data. The quality of the eligible studies was evaluated by Quality Assessment of Diagnostic Accuracy Studies-2, and the result was summarized by Review Manager 5.3. Meta-Disc 1.4 and STATA 16.0 were used in statistical analysis. Heterogeneity of this meta-analysis was calculated. Meta-regression was performed to investigate the potential sources of heterogeneity. Sensitivity analysis and Deek’s funnel plot analysis were conducted to evaluate the stability and publication bias, respectively.

**Results:**

280 studies were initially retrieved through the search strategy, and 10 eligible studies were ultimately included. The random-effects model was selected for data synthesis. The pooled sensitivity, specificity, positive likelihood ratio, negative likelihood ratio, diagnostic odds ratio and the area under the summary receiver operating characteristic curve were 0.92 (95% CI: 0.89–0.94), 0.82 (95% CI: 0.78–0.86), 5.2 (95% CI: 4.1–6.4), 0.10 (95% CI: 0.07–0.13), 54.0 (95% CI: 37.0–77.0) and 0.95 (95% CI: 0.91–0.95). Meta-regression based on study design, country, enrollment and blind method was not statistically significant. This meta-analysis was stable with no obvious publication bias.

**Conclusions:**

The ADNEX model at the 15% cut-off had high diagnostic accuracy in identifying ovarian cancer.

## Introduction

Ovarian cancer is seen as the most aggressive gynecological tumor. The morbidity of ovarian cancer is second to cervical cancer and endometrial cancer, but the mortality ranks first of gynecological tumors. So it is called the “silent killer”. More than 310,000 new cases of ovarian cancer were diagnosed globally in 2020, with nearly 210,000 new deaths, significantly higher than in 2018 (1, 2). The diagnostic reference standard of ovarian cancer depends on pathological examination, but preoperative diagnosis influences doctors’ clinical decisions. Studies indicated that the stage of ovarian cancer is one of the decisive factors affecting the prognosis. For example, the 5-year survival rate of patients with stage IV ovarian cancer was approximately 20%, while patients with stage I ovarian cancer could reach 89% (3). Therefore, improving the accuracy of preoperative diagnosis is of great importance.

Ultrasound is widely used to diagnose and identify ovarian cancer in clinical practice. However, the diagnostic accuracy dramatically depends on the experience of sonographers (4, 5). Ovarian cancer is easily missed or misdiagnosed for its insidious onset and varied image features, especially for inexperienced sonographers. A randomized controlled trial demonstrated that level III (experienced) sonographers are significantly more accurate in diagnosing ovarian cancer than level II (inexperienced) sonographers (6).

To reduce the subjective differences and improve the diagnostic accuracy, International Ovarian Tumor Analysis (IOTA) defines the terms, definitions and measurements used to describe the ultrasonic appearance of ovarian tumors (7). Based on it, IOTA has proposed two logistic regression models (LR1, LR2) and Simple Rules (5, 8). In 2014, a new multiple risk prediction model, Assessment of Different NEoplasias in the adneXa (ADNEX) model, was proposed (9). It consists of six ultrasonic indexes and three clinical indexes. The ultrasonic indexes include the maximum diameter of the lesion, proportion of solid tissue, number of cyst locules (whether more than 10), number of papillary projections (1, 2, 3 or more), presence of acoustic shadows and ascites. Three clinical indexes include age, serum carbohydrate antigen 125 (CA-125) level and category types of centers (oncology center or others). The most significant advantage is that the ADNEX model is the first multi-classification model for ovarian tumors. Based on identifying ovarian cancer from benign tumors, it divides ovarian cancer into four subtypes (borderline, stage I, stages II–IV and metastasis). The overall risk for ovarian cancer and the risk for each subtype can be evaluated simultaneously.

There are limited studies on the diagnosis of the ADNEX model for ovarian cancer because it was published recently. Besides, the cut-off value of the overall risk for ovarian cancer is flexible. In the guideline, the cut-off selected should depend on the centers’ type and the patients’ clinical characteristics. Still, it did not give a recommended cut-off (10). In present studies, 10 and 15% are the most common selected cut-offs to identify the overall risk for ovarian cancer. In the original study, the diagnostic odds at the 10% cut-off and 15% cut-off were 69.2 and 54.7, respectively (9). While in a recent survey, the diagnostic performance of the ADNEX model at the 15% cut-off is better than the 10% cut-off (11). So we have to consider the selection of cut-off value in clinical practice. In a previous meta-analysis, the pooled sensitivity and specificity of the ADNEX model at the 10% cut-off were 0.96 and 0.69 (12). Meanwhile, we noticed that it only included three original studies, and the detailed information of heterogeneity, sensitivity analysis, publication bias was not presented.

To my best knowledge, there is no summary estimate of the 15% cut-off. Therefore, this meta-analysis aimed to discuss the diagnostic accuracy of the ADNEX model at the 15% cut-off of ovarian cancer.

## Materials and Methods

### Search Strategy

We conducted this meta-analysis in compliance with the Preferred Reporting Items for Systematic Reviews and Meta-analyses.

We searched for studies in PubMed, Web of Science, Embase and the Cochrane Library databases published from January 1st, 2014 to February 20th, 2021. A combination of Medical Subject Headings (MeSH) and free text were used to identify related articles. Search terms included “Ovarian Neoplasm”, “Neoplasm, Ovarian”, “Ovarian Neoplasm”, “Ovary Neoplasms”, “Neoplasm, Ovary”, “Neoplasms, Ovary”, “Ovary Neoplasm”, “Neoplasms, Ovarian”, “Ovary Cancer”, “Cancer, Ovary”, “Cancers, Ovary”, “Ovary Cancers”, “Ovarian Cancer”, “Cancer, Ovarian”, “Cancers, Ovarian”, “Ovarian Cancers”, “Cancer of Ovary”, “Cancer of the Ovary”, “Adnexal model”, “ADNEX model” and “Assessment of Different NEoplasias in the adneXa model”. The search was designed to identify all studies on the diagnosis of ovarian cancer with the ADNEX model. Reference lists of the retrieved studies were also screened manually.

### Inclusion and Exclusion Criteria

The inclusion criteria in this meta-analysis were as follows: (1) the subjects were women with ovarian tumors; (2) the diagnostic method was the ADNEX model for ovarian cancer; (3) the reference standard was pathological examination after surgery; (4) retrospective or prospective diagnostic studies; (5) outcome indicators were sensitivity and specificity; and (6) true positive (TP), false positive (FP), true negative (TN), false negative (TN) could be extracted directly or indirectly from the study.

Studies were excluded from these criteria: (1) guidelines, case reports, systematic reviews, and conference studies; (2) lack of original data; (3) duplicate data; (4) unclear cut-off or other cut-off; and (5) inconsistent outcome indicators.

### Data Extraction

The following information was extracted from the eligible studies and drawn into tables: name of the first author, country (Europe *vs.* others), publication year, study design (prospective *vs.* retrospective), enrollment type (consecutive *vs.* unreported), blind method (blind from the reference standard *vs.* unreported), patients’ number, malignant tumors’ number, benign tumors’ number, TP, FP, FN, TN, sensitivity and specificity.

### Quality Assessment

Two investigators (XH and ZW) assessed the quality of the eligible studies by Quality Assessment of Diagnostic Accuracy Studies (QUADAS-2) independently. Every signature question in QUADAS-2 was rated as “yes”, “no” or “unclear”. QUADAS-2 consisted of four assessment sections. Only in one section did all the signature questions answer “yes”, and the corresponding risk of bias was rated as “low”; once any answer was “no”, the risk of bias was rated as “high”; in other cases, the risk of bias was rated as “unclear”. If the evaluations of the two investigators were inconsistent, it would be negotiated by discussion. The final result was presented by Review Manager 5.3 (13).

### Statistical Analysis

Meta-Disc 1.4 and STAT 16.0 were used for statistical analysis (14, 15). Heterogeneity caused by the threshold effect and non-threshold effect was estimated respectively. The threshold effect was identified by the Spearman correlation coefficient between the logit of specificity and logit of 1-specificity. The P-value of the Spearman correlation coefficient >0.05 suggested no threshold effect. The non-threshold effect was evaluated by the inconsistency index (I-squared, I^2^). I^2^ ≦50% indicated no obvious non-threshold effect among the eligible studies. The result of heterogeneity analysis decided the model and effect size selected for data synthesis. The Moses–Shapiro–Littenber model was selected when P-value of the Spearman correlation coefficient <0.05. If so, the simple effect sizes like sensitivity, specificity, positive likelihood ratio (PLR), negative likelihood ratio (NLR) and diagnostic odds ratio (DOR) could not be pooled. Under the premise that P-value of the Spearman correlation coefficient >0.05, the simple effect sizes can be pooled. The fixed-effects model was selected when I^2^ ≦50%, and the random-effects model was selected when I^2^ >50%. Meta-regression analysis was performed to investigate the potential sources of heterogeneity, and the relative diagnostic odds ratio (RDOR) was the evaluation index. RDOR >1 indicated that the studies with this feature had higher diagnostic accuracy than those without, but it was statistically significant only when the corresponding P-value <0.05. Sensitivity analysis was conducted to evaluate the stability of the eligible studies, and Deek’s funnel plot analysis was used to evaluate the publication bias in this meta-analysis.

## Results

### Search Results and Study Characteristics

**Figure 1:**
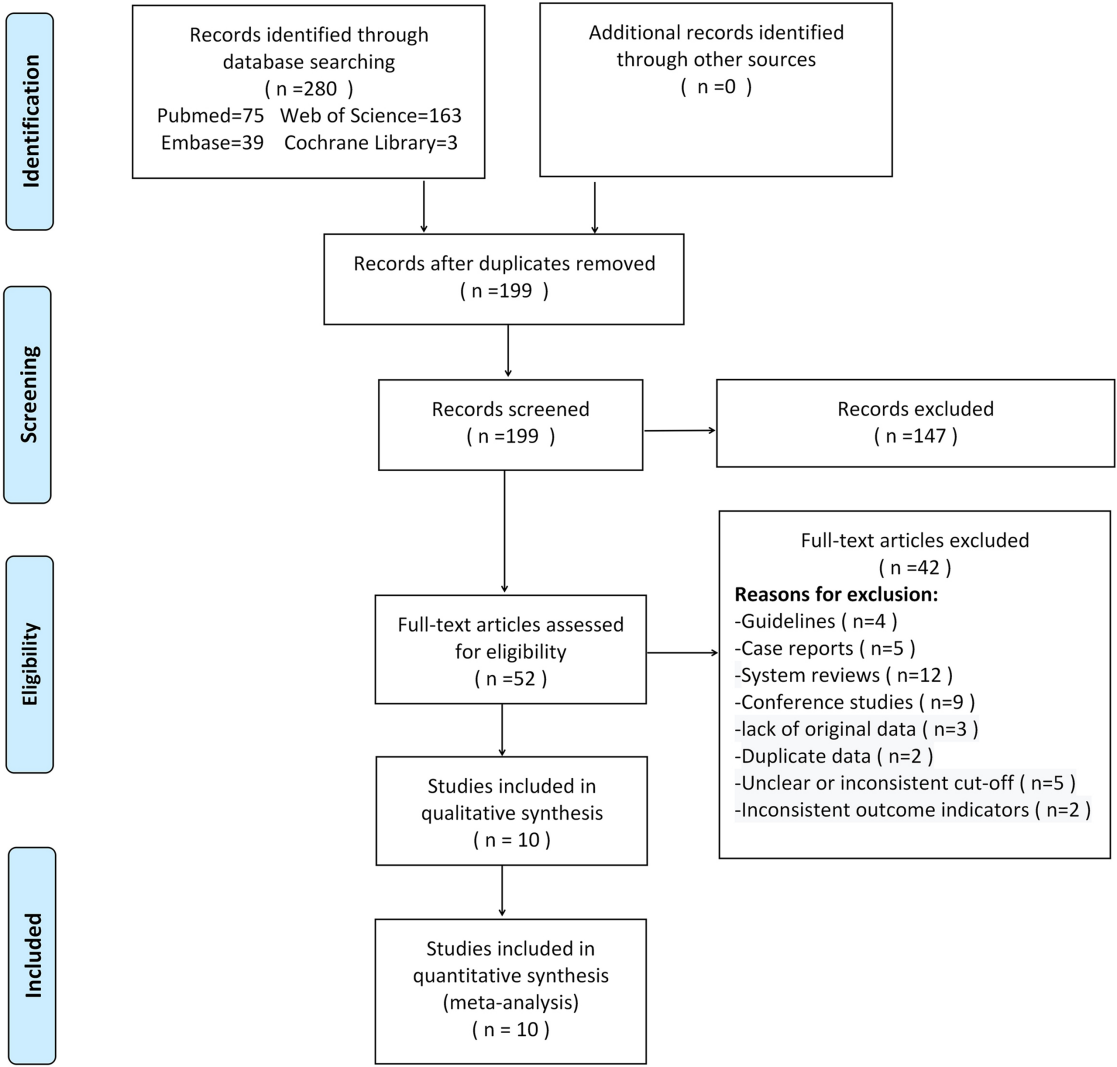
Flow chart of selection process.

**Figure 2:**
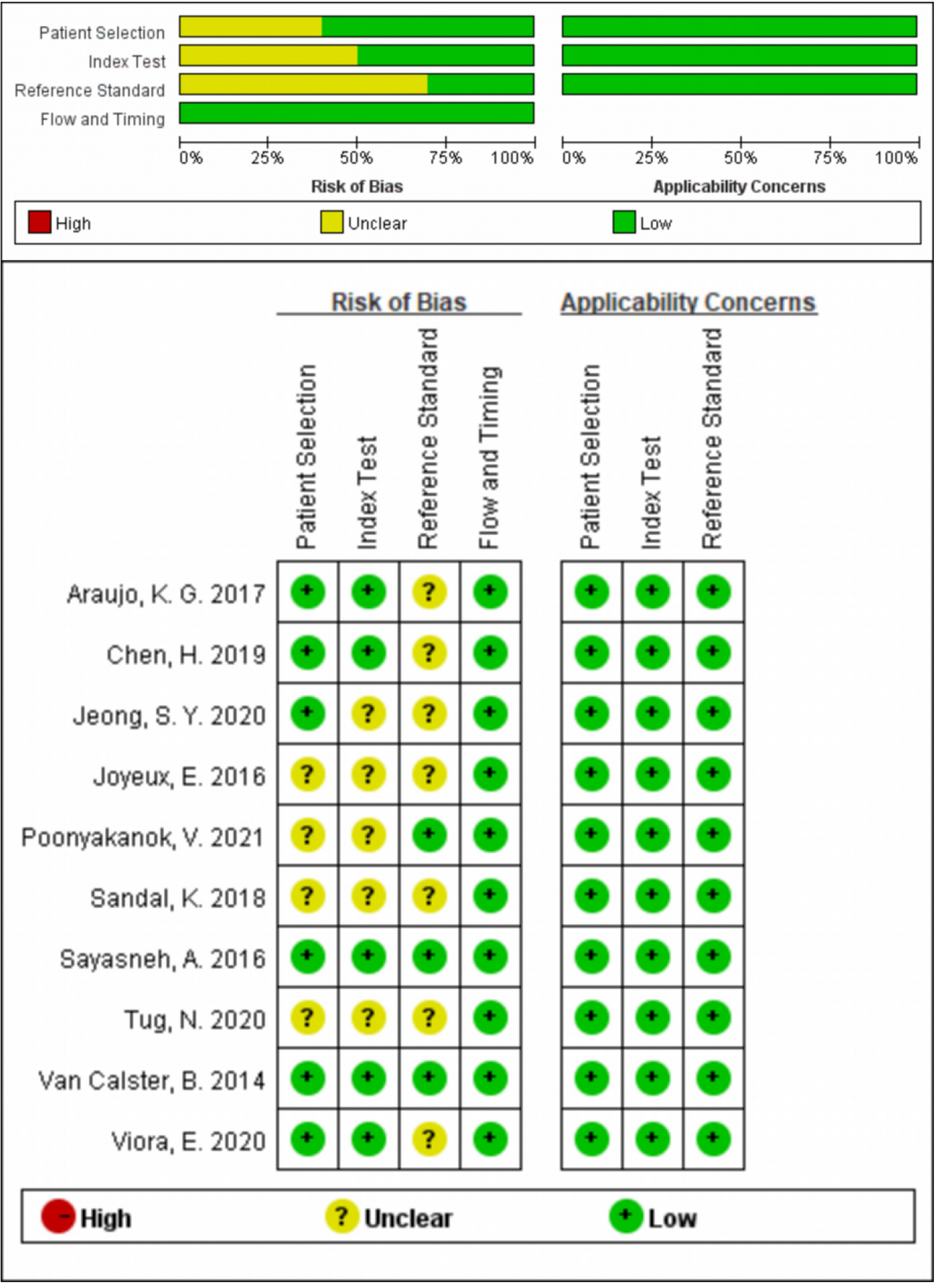
Quality assessment by QUADAS-2.

280 studies were initially retrieved through the search strategy. According to the inclusion and exclusion criteria, a total of 10 studies were ultimately included. There were 5,170 ovarian tumors included, 1,629 were ovarian cancer, and 3,541 were benign tumors. The specific selection process was presented in **Figure 1**. The basic features and parameter data extracted from the 10 eligible studies were shown in **Tables 1**, **2**. The quality assessment by QUADAS-2 was shown in **Figure 2**.

**Table 1:** Basic features of the eligible studies.

Name of the first author	Country	Published year	Enrollment	Study design	Blind method
Araujo (16)	Brazil	2017	consecutive	prospective	blind
Chen (17)	China	2019	consecutive	retrospective	blind
Jeong (18)	Korea	2020	consecutive	prospective	unreported
Joyeux (19)	France	2016	consecutive	retrospective	unreported
Poonyakanok (11)	Thailand	2021	consecutive	prospective	unreported
Sandal (20)	Turkey	2018	unreported	retrospective	unreported
Sayasneh (21)	England	2016	consecutive	prospective	blind
Tug (22)	Turkey	2020	unreported	retrospective	unreported
Van Calster (9)	Belgium	2014	consecutive	prospective	blind
Viora (23)	Italy	2020	consecutive	prospective	blind

**Table 2:** Parameter data extracted from eligible studies.

Name of the first author	Patients’ number	Malignant	Benign	TP	FP	FN	TN	sensitivity	specificity
Araujo, K.G.	131	68	63	62	18	6	45	0.912	0.714
Chen, H.	278	75	203	67	33	8	170	0.893	0.837
Jeong, S.Y.	54	10	44	9	7	1	37	0.9	0.837
Joyeux, E.	284	30	254	26	38	4	216	0.866	0.85
Poonyakanok, V.	357	61	296	60	28	1	268	0.984	0.905
Sandal, K.	191	53	138	50	38	3	100	0.943	0.725
Sayasneh, A.	610	182	428	172	106	10	322	0.944	0.752
Tug, N.	285	26	259	22	26	4	233	0.846	0.9
Van Calster, B.	2,403	980	1,423	923	324	57	1,099	0.942	0.772
Viora, E.	577	144	433	126	80	18	353	0.875	0.815

### Meta-Analysis

#### Heterogeneity Analysis

**Figure 3:**
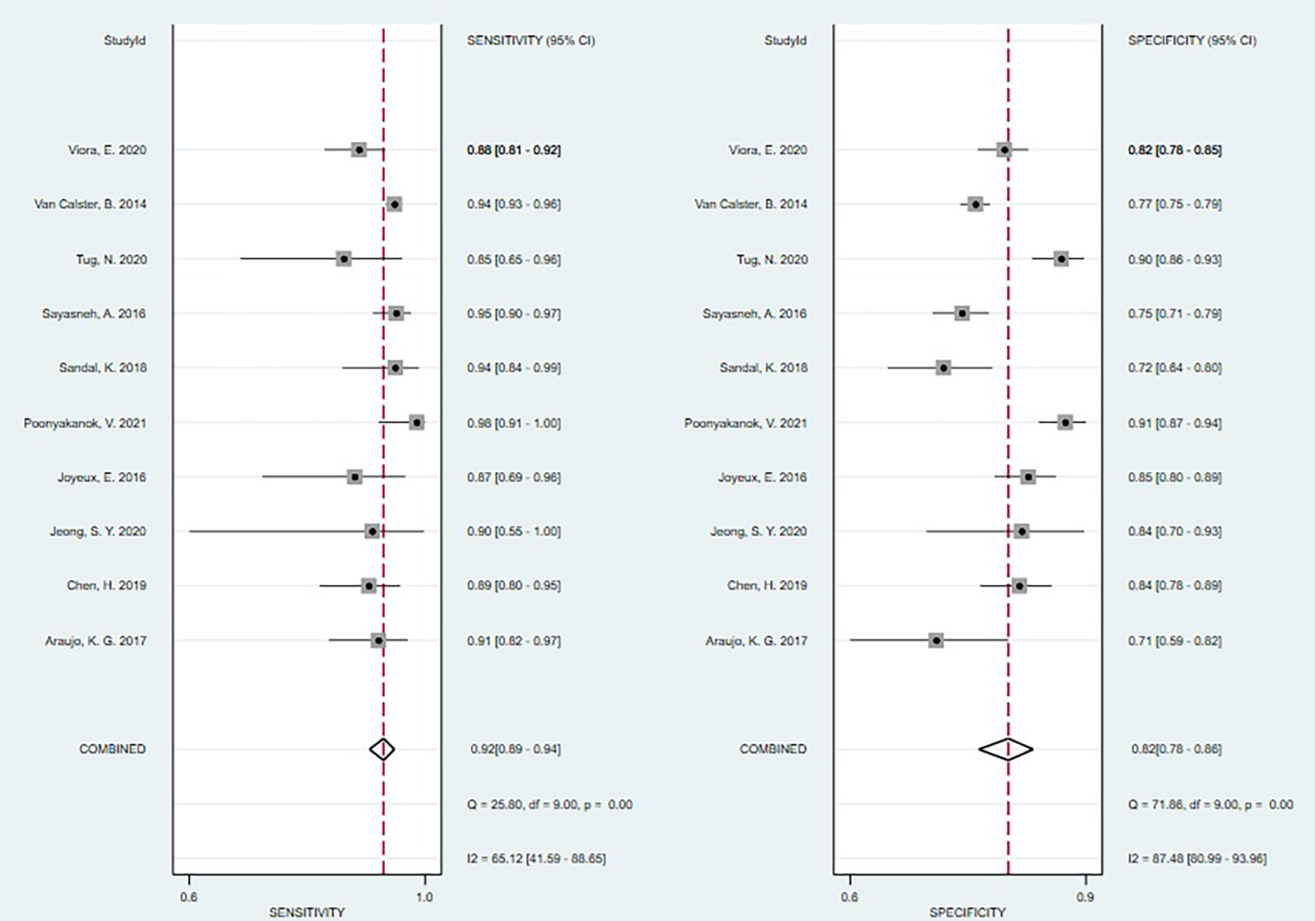
Forest plots for the diagnostic accuracy.

The P-value of the Spearman correlation coefficient was 0.365 (>0.05), which indicated no threshold effect in this analysis. I^2^ = 65.12% (>50%) meant the existence of non-threshold effect heterogeneity (**Figure 3**).

#### Data Synthesis

According to the result of heterogeneity analysis, there was no threshold effect but non-threshold effect in this meta-analysis. The random-effects model was selected to pool the effect sizes. The pooled sensitivity, specificity, PLR, NLR, DOR and the area under the curve (AUC) were 0.92 (95% CI: 0.89–0.94), 0.82 (95% CI: 0.78–0.86), 5.20 (95% CI: 4.10–6.40), 0.10 (95% CI: 0.07–0.13), 54.0 (95% CI: 37.0–77.0), 0.95 (95% CI: 0.91–0.95).

#### Meta-Regression

According to the features of the 10 eligible studies, meta-regression was performed based on the following factors: study design, country, enrollment, blind method. As shown in **Table 3**, the RDOR of country and blind method was less than 1.0, which meant the diagnostic accuracy was not influenced by country or blind method. The RDOR of study design and enrollment was greater than 1.0, but the P-value was greater than 0.05. It meant the diagnostic accuracy of prospective studies was higher than retrospective studies, and the diagnostic accuracy of studies with consecutive enrollment was higher than non-consecutive enrollment. However, neither was statistically significant. Therefore, none of the evaluated factors could explain the heterogeneity in this meta-analysis.

**Table 3:** Meta-regression analysis.

Factor		Coeff.	P	RDOR	95%CI
Study design					
	Prospective *vs.* Retrospective	0.5861	0.6703	1.31	0.26–6.66
Country					
	Europe *vs.* Others	−0.305	0.5559	0.74	0.20–2.75
Enrollment					
	Consecutive *vs.* Unreported	0.300	0.6687	1.35	0.22–8.19
Blind method					
	Blind *vs.* Unreported	0.645	0.2847	0.52	0.12–2.24

#### Sensitivity Analysis

**Figure 4:**
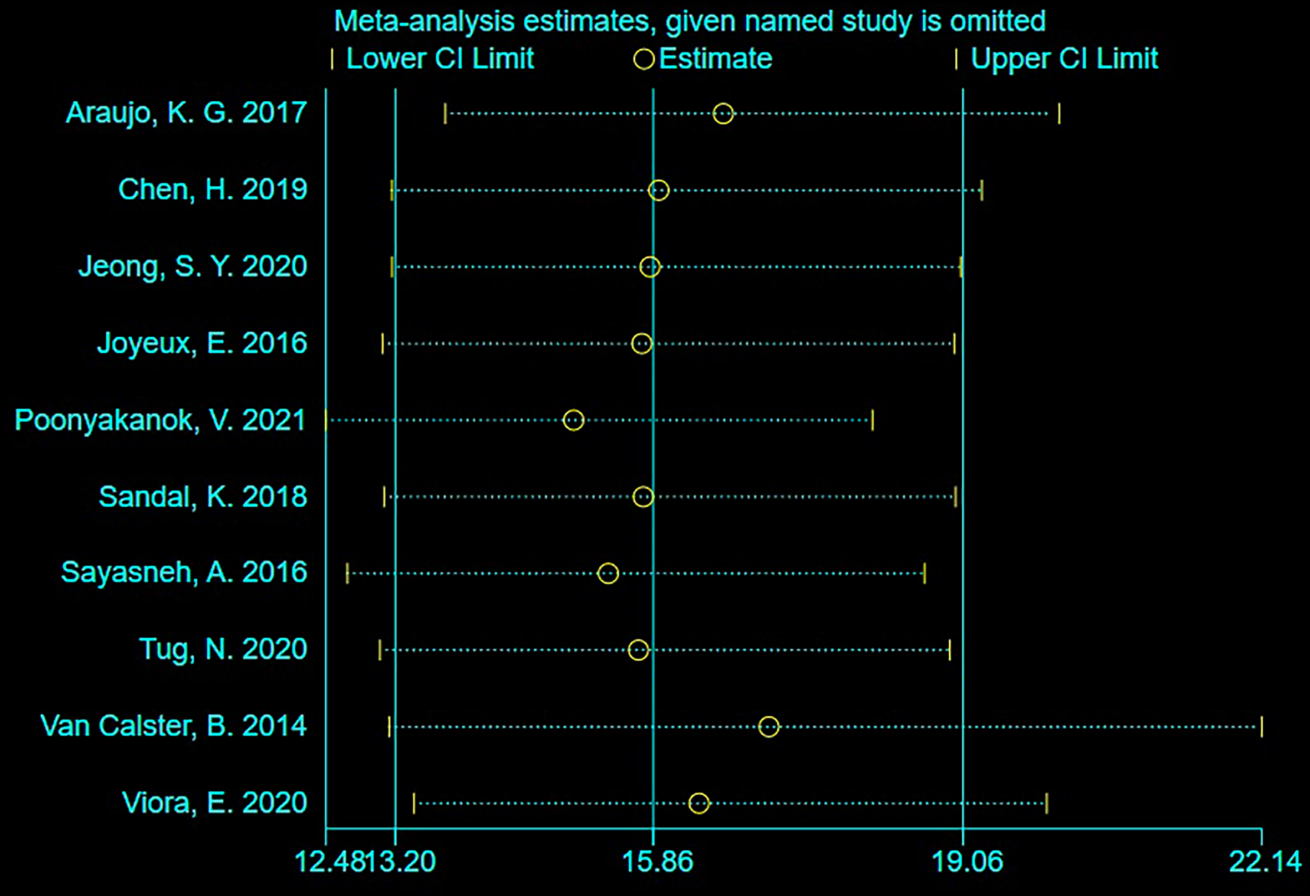
Sensitivity analysis.

As shown in **Figure 4**, sensitivity analysis was conducted after the study was excluded one by one, and the overall sensitivity and specificity were not significantly changed. It suggested that the combined effect sizes in this meta-analysis were relatively stable without over-dependence on any single study.

#### Deek’s Funnel Plot Analysis

**Figure 5:**
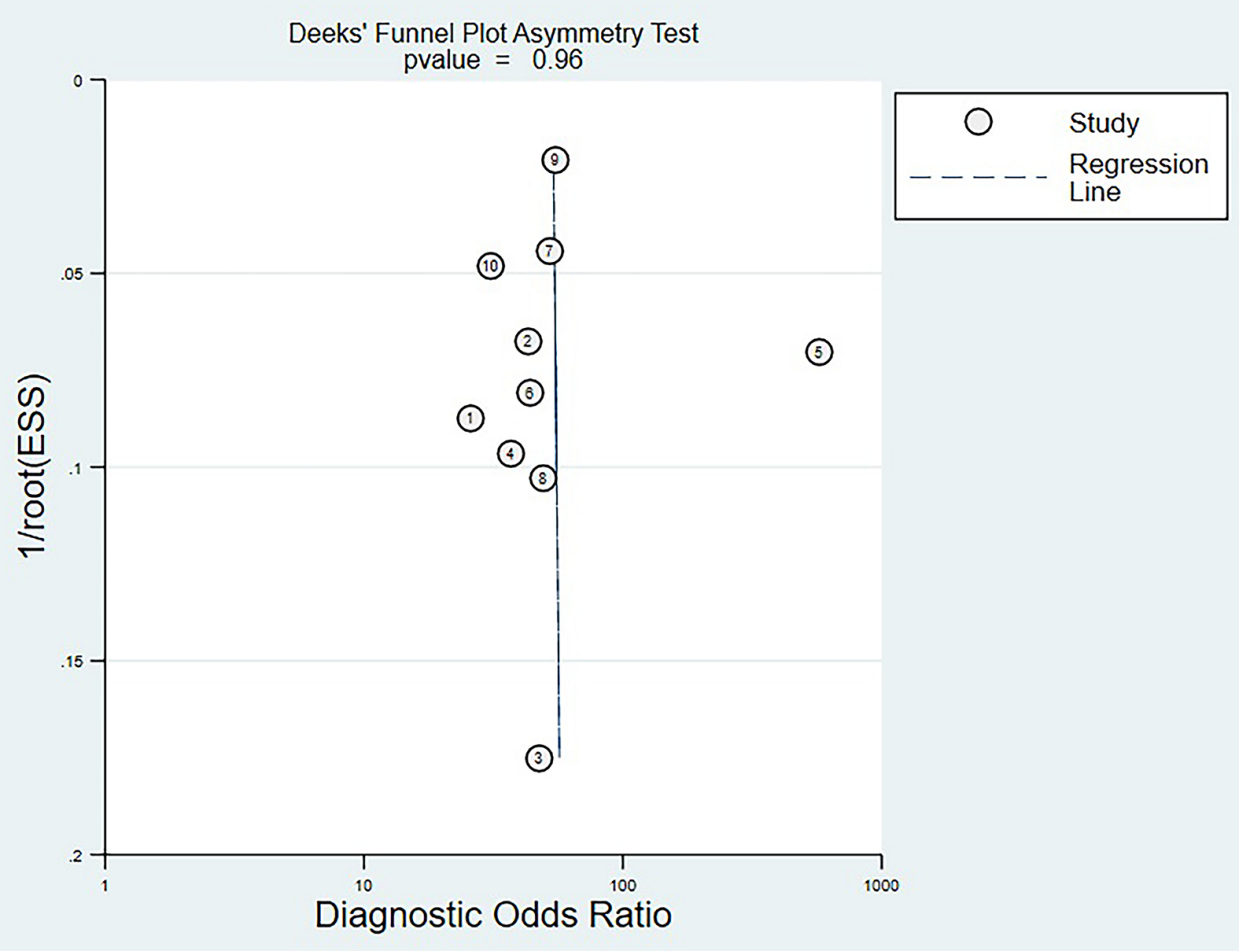
Deek's funnel plot analysis.

Deek’s funnel plot analysis showed that scattered points were evenly distributed on both sides of the regression line, and P-value was 0.96 (>0.05). There was no significant publication bias among the eligible studies (**Figure 5**).

## Discussion

### Main Findings

In this meta-analysis, a total of 10 studies on the preoperative diagnosis of the ADNEX model at the 15% cut-off for ovarian cancer were eligible. The pooled sensitivity, specificity, PLR, NLR, DOR and AUC were 0.92 (95% CI: 0.89–0.94), 0.82 (95% CI: 0.78–0.86), 5.2 (95% CI: 4.1–6.4), 0.10 (95% CI: 0.07–0.13), 54.0 (95% CI: 37.0–77.0), 0.95 (95% CI: 0.91–0.95), respectively. It meant that the ADNEX model at the 15% cut-off had high specificity while ensuring sensitivity. Higher sensitivity meant identifying more suspicious patients and referring them for further examinations. It helped minimize the risk of delaying treatment for suspicious patients. Higher specificity meant reducing the false positive rate. It was of great significance for optimizing the allocation of medical resources and reducing the unnecessary costs of patients.

There was no threshold effect in this meta-analysis, but the non-threshold effect heterogeneity could not be ignored. Meta-regression was conducted to investigate the potential sources of heterogeneity. In general, studies with the prospective design, consecutive enrollment and blind method could substantially reduce the subjective influence from the researchers. From the previous report, the incidence of ovarian cancer varies in different regions, and it is significantly higher in European countries than in others (3). Therefore, study design, country, enrollment and blind method were selected as the potential factories causing the non-threshold effect heterogeneity in this meta-analysis. However, none of the evaluated factors were the sources of heterogeneity. And the result of sensitivity analysis and publication bias risk test showed that this meta-analysis was stable and reliable.

### Comparison With Other Models

There are many prediction models for the diagnosis of ovarian cancer. At present, Risk of Malignancy Index (RMI) is the most widely used model and recommended by most oncology centers. Studies showed that RMI I has the highest diagnostic accuracy among RMI I–IV (24, 25). While in a recent meta-analysis, the DOR of RMI I was 33.0, which was not satisfactory (26). In our meta-analysis, the DOR of the ADNEX model at the 15% cut-off was 54.0. So we thought the ADNEX model at the 15% cut-off was better than RMI. Another multi-center study also verified that the Net Benefit of the ADNEX model is higher than Risk of Ovarian Malignancy Algorithm (ROMA), RMI and LR2 (27). Meys et al. (28) validated the diagnostic accuracy of subjective assessment and four frequently used models (Simple Rules, LR2, RMI and the ADNEX model), and the results showed that the ADNEX model performs better than the other three models, but the subjective assessment of expert sonographers still performs the best. However, Viora et al. (23) and Epstein et al. (29) found the opposite. They pointed out that the diagnostic accuracy of the ADNEX model was equal to, or even more accurate than the subjective assessment of expert sonographers. The ADNEX model aims at helping inexperienced sonographers and gynecologists classify patients for appropriate treatment, not as a substitute for expert evaluation (30). Meanwhile, we must be aware that expert sonographers are not always available. Furthermore, compared with the ADNEX model at the 10% cut-off, the sensitivity of this model at the 15% cut-off decreased slightly (10%: 0.96, 15%: 0.92) but specificity (10%: 0.69, 15%: 0.82) increased significantly (12).

Therefore, the ADNEX model deserved to be promoted in clinical practice.

### Further Optimization

The model proposed by Stukan (31) inspired us to think about the further optimization of the ADNEX model. This concise model only includes three indexes: solid areas, color score and the level of D-dimer. However, the diagnostic accuracy is comparable to the ADNEX model. Thus, we had reasons to believe that the ADNEX model could be further optimized.

Firstly, we should consider the selection of tumor markers in the ADNEX model. Studies demonstrated that the absence of CA-125 has no significant effect on the diagnostic performance of the ADNEX model (11, 17, 21). CA-125 is not specific to ovarian cancer, and it can increase in benign lesions, such as endometriosis and uterine fibroids (32–34). Human epididymal protein-4 (HE-4) has become a novel tumor marker for ovarian cancer (35). Simona et al. (36) compared the diagnostic accuracy of HE-4 and CA-125 for ovarian cancer, and the result showed the PLR and NLR for HE-4 were 13.0 and 0.23, but 4.2 and 0.27 for CA-125. Some other studies also verified that HE-4 is more valuable than CA-125 for ovarian cancer (37–39). However, the study of McKendry et al. (40) indicated that CA-125 performs the best in premenopausal women. So the selection of tumor markers needs to be validated by more studies.

Secondly, more researchers have noticed that a high D-dimer level is an important diagnostic marker for ovarian cancer patients. The level of D-Dimer has traditionally been used to assess the risk of thrombosis in patients with ovarian cancer (41, 42). While studies demonstrated that the D-dimer level in a patient with ovarian cancer is significantly higher than that with benign tumors, and the D-dimer level in a patient with stage III–IV ovarian cancer is higher than that with stage I–II ovarian cancer (43–45). Another study showed that the D-dimer level increased in 73% stage I ovarian cancer patients, whereas CA-125 increased in only 33%, so D-dimer is more sensitive in stage I ovarian cancer patients (46). Moreover, a previous study showed that D-dimer is a useful marker in differentiating ovarian cancer and endometriosis, with a sensitivity of 93.2% and a specificity of 87.5% (47). Therefore, the inclusion of D-Dimer was expected to improve the accuracy in identifying the subtypes of ovarian cancer.

Thirdly, Color Doppler is used widely in clinical practice, but the ADNEX model only includes two-dimensional morphological ultrasonic indexes. The blood flow signals within the ovarian tumor are also important to identify the nature of ovarian tumors (38). As we know, the blood flow signals of ovarian cancer are abundant and disorderly, and the resistance index (RI) is usually less than 0.5. Researchers found that the sensitivity and specificity of Color Doppler for ovarian cancer were 71.88 and 84.29 (48). Although different ultrasonic instruments may have certain differences in the sensitivity to blood flow signals, most instruments are highly sensitive and can meet the diagnostic requirement. Besides, another research indicated the diagnosis of ovarian cancer by Color Doppler is in good consistency within and between observers (49).

### Strengths and Limitations

The strengths of this meta-analysis were summarized as follows. Firstly, this was the first summary estimate of the diagnostic accuracy of the ADNEX model at the 15% cut-off value. Secondly, the included studies were of high quality. Among the 10 eligible studies, six were prospective studies, eight were consecutive enrollment studies, and five were blind from reference standard. Thirdly, this meta-analysis was stable without significant publication bias.

This analysis had some limitations. Firstly, there were only 10 eligible studies included in this meta-analysis. More studies are needed to verify the diagnostic performance of it. Secondly, the limitation of territory should be taken into account. Five studies were carried out in Europe and four in Asian countries, but only one study in South America. In fact, the ADNXE model is not implemented widely in America (30). So it was hard to evaluate the diagnostic performance globally. Thirdly, the most significant advantage of the ADNEX model is the division of four subtypes of ovarian cancer. While in this meta-analysis, complete diagnostic data in 2 × 2 tables of the four subtypes could not be extracted in eight studies. So we only discussed the diagnostic accuracy in the identification of ovarian cancer from benign tumors. The remaining two studies indicated that the ADNEX model is less effective in differentiating stage I ovarian cancer from borderline tumors and stage II–IV ovarian cancer from metastasis (17, 23, 28). Still, future studies are needed to assess the diagnostic accuracy in different subtypes.

## Conclusion

In summary, the ADNEX model at the 15% cut-off was in high diagnostic accuracy for identifying the risk of ovarian cancer, which should be accepted and promoted more widely. At the same time, more studies on the diagnostic accuracy for different subtypes and the optimization of this model deserve exploring and expecting.

## Data Availability Statement

The original contributions presented in the study are included in the article/supplementary material. Further inquiries can be directed to the corresponding author.

## Author Contributions

XH, HL, ZW, and MZ: study design and manuscript review. XH and ZW: literature screen, data extraction, quality assessment. HL and MZ: inconsistency negotiation and quality control. XH and MZ: statistical analysis and manuscript composing. All authors contributed to the article and approved the submitted version.

## Conflict of Interest

The authors declare that the research was conducted in the absence of any commercial or financial relationships that could be construed as a potential conflict of interest.
